# Supplementary material for: Validation-based model selection for 13C metabolic flux analysis with uncertain measurement errors
Source: PLoS Comput Biol. 2022 Apr 11;18(4):e1009999. doi: 10.1371/journal.pcbi.1009999 (PMC9022838; doi:10.1371/journal.pcbi.1009999)
Supplement: S1 Algorithm — The model selection algorithm takes a set of model structures and a set of data as inputs and selects the most appropriate model structure based on the sub type (A-D) and the data. Subtype A selects the model structure that yields the smallest summed squared residuals (SSR) given the entire data set. Subtype B selects the first/simplest model structure that can pass a χ2-test. Subtype C selects the model structure that passes a χ2-test with the largest margin. Finally, subtype D selects the model structure that yields the lowest SSR with respect to a validation subset of the data. (DOCX) [file pcbi.1009999.s008.docx]

# Algorithm

### Algorithm 1: Model selection algorithms, type A-D combined

Input: A set of model structures $\left\{ \mathcal{M}_{1}\ldots\mathcal{M}_{n} \right\}$, a set of data $D$.

*if* algorithm sub-type is A-C, which do not use validation data, the entire data set $D$ is used for estimation: $D=D^{est}$.

*if* algorithm sub-type is D, the data$D$ is divided into two datasets, estimation data $D^{est}$ and validation data $D^{val}$: $D= \{D^{est},D^{val}\}$.

*for* all $\mathcal{M}_{j}$, $j=1\ldots n$

Fit model $\mathcal{M}_{j}$ to estimation data

$u_{j}^{*}=\arg\min_{u} SSR^{est}\left( \mathcal{M}_{j},D^{\mathrm{est}},u \right) s.t. g_{j}\left( \theta\right)\geq0 \forall j$

end *for*

1. *(“Smallest SSR”)* Find the model structure $\mathcal{M'}$ with the lowest $SSR^{Est}$, with respect to $u_{j}^{*}$ and $D^{Est}$. $\mathcal{M}^{'}=\arg\min_{\mathcal{M}_{j}} SSR^{Est}(\mathcal{M}_{j},D^{Est},u_{j}^{*})$.
2. *(“First* $\chi^{2}$*”)* Find the model structure $\mathcal{M'}$ which is the simplest $\mathcal{M}_{j}$ to pass a $\chi^{2}$ test, i.e. $SSR^{Est}<\chi^{2,cuminv}(p=0.95,N^{est}-\#u_{j})$.
3. *(“Best* $\chi^{2}$*”)* Find the model structure $\mathcal{M'}$ with the lowest difference between $SSR^{Est}$ and the $\chi^{2}$-threshold, with respect to $u_{j}^{*}$ and $D^{Est}$
   - 1. $\mathcal{M}^{'}=\arg\min_{\mathcal{M}_{j}} (SSR^{Est}\left( \mathcal{M}_{j},D^{Est},u_{j}^{*} \right)-\chi^{2,cuminv}(p=0.95,N-\#u_{j}))$.
4. *(“Validation-based selection”)* Find the model structure $\mathcal{M'}$ with the lowest $SSR^{val}$, with respect to $u_{j}^{*}$ and $D^{val},$ $\mathcal{M}^{'}=\arg\min_{\mathcal{M}_{j}} SSR^{val}(\mathcal{M}_{j},D^{val},u_{j}^{*})$.

Output: Model structure $\mathcal{M}^{'}$ that offers the most accurate description of the target system.
