# Supplementary material for: Validation-based model selection for 13C metabolic flux analysis with uncertain measurement errors
Source: PLoS Comput Biol. 2022 Apr 11;18(4):e1009999. doi: 10.1371/journal.pcbi.1009999 (PMC9022838; doi:10.1371/journal.pcbi.1009999)
Supplement: S1 Table — The table contains a detailed breakdown of the reactions that are included in the TCA-models. In general, the model structures are derived from the most complex model structure M7 by successively removing and combining reactions and thus make each successive model structure simpler. (DOCX) [file pcbi.1009999.s009.docx]

**Table S1: Breakdown of reactions for the TCA-cycle models.** The table contains a detailed breakdown of the reactions that are included in the TCA-models. In general, the model structures are derived from the most complex model structure $\mathcal{M}_{7}$ by successively removing and combining reactions and thus make each successive model structure simpler.

| Reaction | Reversable | Included in model structure |
| --- | --- | --- |
| succoa_m + adp_m + pi_m = succ_m + atp_m + coa_m | Yes | ℳ_7_,ℳ_6_,ℳ_5_,ℳ_4_,ℳ_3_,ℳ_2_,ℳ_1_ |
| akg_m + coa_m + nad_m = succoa_m + co2_m + nadh_m | No | ℳ_7_,ℳ_6_,ℳ_5_,ℳ_4_,ℳ_3_,ℳ_2_ |
| icit_m + nad_m = akg_m + co2_m + nadh_m | Yes | ℳ_7_,ℳ_6_,ℳ_5_ |
| acnt_m = icit_m | Yes | ℳ_7_,ℳ_6_,ℳ_5_ |
| co2_m = co2_c | No | ℳ_7_,ℳ_6_,ℳ_5_ |
| gln_L_c = gln_L_m | Yes | ℳ_7_,ℳ_6_,ℳ_5_,ℳ_4_,ℳ_3_,ℳ_2_,ℳ_1_ |
| glu_L_m + h2o_m + nad_m = akg_m + h_m + nadh_m + nh4_m | Yes | ℳ_7_,ℳ_6_,ℳ_5_,ℳ_4_,ℳ_3_,ℳ_2_,ℳ_1_ |
| gln_L_m + h2o_m = glu_L_m + nh4_m | Yes | ℳ_7_,ℳ_6_,ℳ_5_,ℳ_4_,ℳ_3_,ℳ_2_,ℳ_1_ |
| nh4_m = nh4_c | No | ℳ_7_,ℳ_6_,ℳ_5_,ℳ_4_,ℳ_3_,ℳ_2_,ℳ_1_ |
| succ_m + q10_m = fum_m + q10h2_m | No | ℳ_7_,ℳ_6_,ℳ_5_,ℳ_4_,ℳ_3_,ℳ_2_ |
| fum_m + h2o_m = mal_L_m | Yes | ℳ_7_,ℳ_6_,ℳ_5_,ℳ_4_,ℳ_3_,ℳ_2_,ℳ_1_ |
| mal_L_m + nad_m = oaa_m + h_m + nadh_m | Yes | ℳ_7_,ℳ_6_,ℳ_5_,ℳ_4_,ℳ_3_,ℳ_2_,ℳ_1_ |
| cit_m = acnt_m | Yes | ℳ_7_,ℳ_6_,ℳ_5_,ℳ_4_,ℳ_3_,ℳ_2_,ℳ_1_ |
| oaa_m + accoa_m + h2o_m = cit_m + coa_m + h_m | No | ℳ_7_ |
| pyr_m + coa_m + nad_m = accoa_m + co2_m + nadh_m | No | ℳ_7_ |
| mal_L_m + nad_m = pyr_m + co2_m + nadh_m | No | ℳ_7_,ℳ_6_,ℳ_5_,ℳ_4_,ℳ_3_,ℳ_2_,ℳ_1_ |
| pyr_c = pyr_m | Yes | ℳ_7_,ℳ_6_,ℳ_5_,ℳ_4_,ℳ_3_,ℳ_2_,ℳ_1_ |
| co2_m + h2o_m = hco3_m + h_m | No | ℳ_7_,ℳ_6_ |
| hco3_m + pyr_m + atp_m = oaa_m + adp_m + h_m + pi_m | No | ℳ_7_,ℳ_6_ |
| ac_m + atp_m + coa_m = accoa_m + amp_m + ppi_m | No | ℳ_7_ |
| amp_m + atp_m = adp_m + adp_m | No | ℳ_7_ |
| h2o_m + ppi_m = h_m + 2 pi_m | No | ℳ_7_ |
| cit_m = cit_c | Yes | ℳ_7_,ℳ_6_,ℳ_5_,ℳ_4_,ℳ_3_,ℳ_2_,ℳ_1_ |
| oaa_c = oaa_m | Yes | ℳ_7_,ℳ_6_,ℳ_5_,ℳ_4_,ℳ_3_,ℳ_2_,ℳ_1_ |
| cit_c + atp_c + coa_c = oaa_c + accoa_c + adp_c + pi_c | No | ℳ_7_,ℳ_6_,ℳ_5_,ℳ_4_ |
| accoa_c + h2o_c = ac_c + coa_c + h_c | No | ℳ_7_,ℳ_6_,ℳ_5_,ℳ_4_ |
| 5 h_m + nadh_m + q10_m =4 h_ims_m + nad_m + q10h2_m | No | ℳ_7_,ℳ_6_,ℳ_5_,ℳ_4_,ℳ_3_,ℳ_2_ |
| 2 ficytC_m +2 h_m + q10h2_m = 2 focytC_m + 4 h_ims_m + q10_m | No | ℳ_7_,ℳ_6_,ℳ_5_,ℳ_4_,ℳ_3_,ℳ_2_ |
| 4 focytC_m + 7 h_m + o2_m = 4 ficytC_m + 2 h2o_m + 4 h_ims_m | No | ℳ_7_,ℳ_6_,ℳ_5_,ℳ_4_,ℳ_3_,ℳ_2_ |
| adp_m + 4 h_ims_m + pi_m = atp_m + 3 h_m + h2o_m | No | ℳ_7_,ℳ_6_,ℳ_5_,ℳ_4_,ℳ_3_,ℳ_2_ |
| h_c = h_m | No | ℳ_7_,ℳ_6_,ℳ_5_,ℳ_4_,ℳ_3_,ℳ_2_ |
| h2o_m = h2o_c | No | ℳ_7_,ℳ_6_,ℳ_5_,ℳ_4_,ℳ_3_,ℳ_2_ |
| o2_c = o2_m | No | ℳ_7_,ℳ_6_,ℳ_5_,ℳ_4_,ℳ_3_,ℳ_2_ |
| pi_c = pi_m | No | ℳ_7_,ℳ_6_,ℳ_5_,ℳ_4_,ℳ_3_,ℳ_2_ |
| adp_c + atp_m = adp_m + atp_c | No | ℳ_7_,ℳ_6_,ℳ_5_,ℳ_4_,ℳ_3_,ℳ_2_ |
| atp_c + h2o_c = adp_c + energy_c + h_c + pi_c | No | ℳ_7_,ℳ_6_,ℳ_5_,ℳ_4_,ℳ_3_,ℳ_2_ |
| asp_L_m + akg_m = oaa_m + glu_L_m | Yes | ℳ_7_,ℳ_6_,ℳ_5_,ℳ_4_,ℳ_3_,ℳ_2_,ℳ_1_ |
| cit_ex = cit_c | No | ℳ_7_,ℳ_6_,ℳ_5_,ℳ_4_,ℳ_3_,ℳ_2_,ℳ_1_ |
| akg_c = akg_m | Yes | ℳ_7_,ℳ_6_,ℳ_5_,ℳ_4_,ℳ_3_,ℳ_2_,ℳ_1_ |
| asp_L_m + glu_L_c + h_c = asp_L_c + glu_L_m + h_m | No | ℳ_7_,ℳ_6_,ℳ_5_,ℳ_4_,ℳ_3_,ℳ_2_,ℳ_1_ |
| asp_L_c + akg_c = oaa_c + glu_L_c | Yes | ℳ_7_,ℳ_6_,ℳ_5_,ℳ_4_,ℳ_3_,ℳ_2_,ℳ_1_ |
| mal_L_c + nad_c = oaa_c + h_c + nadh_c | Yes | ℳ_7_,ℳ_6_,ℳ_5_,ℳ_4_,ℳ_3_ |
| mal_L_c = mal_L_m | Yes | ℳ_7_,ℳ_6_,ℳ_5_,ℳ_4_,ℳ_3_,ℳ_2_,ℳ_1_ |
| fum_m = fum_c | Yes | ℳ_7_,ℳ_6_,ℳ_5_,ℳ_4_,ℳ_3_,ℳ_2_,ℳ_1_ |
| succ_m = succ_c | Yes | ℳ_7_,ℳ_6_,ℳ_5_,ℳ_4_,ℳ_3_,ℳ_2_ |
| fum_c + succ_m = fum_m + succ_c | Yes | ℳ_7_,ℳ_6_,ℳ_5_,ℳ_4_,ℳ_3_,ℳ_2_ |
| fum_c + mal_L_m = fum_m + mal_L_c | Yes | ℳ_7_,ℳ_6_,ℳ_5_,ℳ_4_,ℳ_3_,ℳ_2_,ℳ_1_ |
| fum_c + h2o_c = mal_L_c | Yes | ℳ_7_,ℳ_6_,ℳ_5_,ℳ_4_,ℳ_3_,ℳ_2_,ℳ_1_ |
| succ_c + coa_c + atp_c = succoa_c + adp_c + pi_c | No | ℳ_7_,ℳ_6_,ℳ_5_,ℳ_4_,ℳ_3_,ℳ_2_ |
| pyr_m + oaa_m = cit_m + co2_m | No | ℳ_6_,ℳ_5_,ℳ_4_,ℳ_3_,ℳ_2_,ℳ_1_ |
| akg_m = fum_m + co2_m | No | ℳ_1_ |
| acnt_m = akg_m + co2_m | No | ℳ_4_,ℳ_3_,ℳ_2_,ℳ_1_ |
