# Supplementary material for: Validation-based model selection for 13C metabolic flux analysis with uncertain measurement errors
Source: PLoS Comput Biol. 2022 Apr 11;18(4):e1009999. doi: 10.1371/journal.pcbi.1009999 (PMC9022838; doi:10.1371/journal.pcbi.1009999)
Supplement: S2 Table — The full parameter vector for the polynomial, linear and metabolic flux analysis model examples are given by the respective columns. (DOCX) [file pcbi.1009999.s010.docx]

***Table S2:*** *A summary of the parameter values that were used to generate the simulated data for the three different examples that are used in the manuscript. The full parameter vector for the polynomial, linear and metabolic flux analysis model examples are given by the respective columns.*

| *Parameter index* | *Polynomial parameter values* | *Linear system parameter values* | *MFA example flux values* |
| --- | --- | --- | --- |
| θ_1_ | 0.0010 | *5* | 102.20 |
| θ_2_ | -0.0338 | *8* | 99.25 |
| θ_3_ | 0.4708 | *2* | 2.95 |
| θ_4_ | -3.2936 |  | 2.53 |
| θ_5_ | 11.9932 |  | 4.85 |
| θ_6_ | -20.8493 |  | 4.42 |
| θ_7_ | 12.9469 |  | 10.95 |
| θ_8_ | 2.2239 |  | 10.53 |
| θ_9_ |  |  | 1.99 |
| θ_10_ |  |  | 1.56 |
| θ_11_ |  |  | 0.85 |
| θ_12_ |  |  | 2.95 |
| θ_13_ |  |  | 201.85 |
| θ_14_ |  |  | 199.00 |
| θ_15_ |  |  | 0.01 |
| θ_16_ |  |  | 2.30 |
| θ_17_ |  |  | 43.78 |
| θ_18_ |  |  | 41.26 |
| θ_19_ |  |  | 1.40 |
| θ_20_ |  |  | 11.42 |
| θ_21_ |  |  | 0.00 |
| θ_22_ |  |  | 12.82 |
| θ_23_ |  |  | 14.75 |
| θ_24_ |  |  | 4.46 |
| θ_25_ |  |  | 214.11 |
| θ_26_ |  |  | 199.00 |
| θ_27_ |  |  | 11.26 |
| θ_28_ |  |  | 11.26 |
| θ_29_ |  |  | 2.49 |
| θ_30_ |  |  | 5.44 |
| θ_31_ |  |  | 2.72 |
| θ_32_ |  |  | 10.65 |
| θ_33_ |  |  | 15.00 |
| θ_34_ |  |  | 12.39 |
| θ_35_ |  |  | 2.72 |
| θ_36_ |  |  | 13.60 |
| θ_37_ |  |  | 13.60 |
| θ_38_ |  |  | 2.34 |
| θ_39_ |  |  | 0.00 |
| θ_40_ |  |  | 0.00 |
| θ_41_ |  |  | 0.97 |
| θ_42_ |  |  | 22.04 |
| θ_43_ |  |  | 22.04 |
| θ_44_ |  |  | 0.00 |
| θ_45_ |  |  | 99.71 |
| θ_46_ |  |  | 99.71 |
| θ_47_ |  |  | 202.85 |
| θ_48_ |  |  | 199.00 |
| θ_49_ |  |  | 1.32 |
| θ_50_ |  |  | 0.08 |
| θ_51_ |  |  | 200.29 |
| θ_52_ |  |  | 199.00 |
| θ_53_ |  |  | 4.19 |
| θ_54_ |  |  | 0.40 |
| θ_55_ |  |  | 195.21 |
| θ_56_ |  |  | 199.00 |
| θ_57_ |  |  | 203.98 |
| θ_58_ |  |  | 199.00 |
| θ_59_ |  |  | 199.10 |
| θ_60_ |  |  | 199.00 |
| θ_61_ |  |  | 0.00 |
