# Supplementary material for: Validation-based model selection for 13C metabolic flux analysis with uncertain measurement errors
Source: PLoS Comput Biol. 2022 Apr 11;18(4):e1009999. doi: 10.1371/journal.pcbi.1009999 (PMC9022838; doi:10.1371/journal.pcbi.1009999)
Supplement: Attachment — Submitted filename: Response_to_reviewers.docx [file pcbi.1009999.s011.docx]

**–Reviewer #1: The authors propose an approach to select which metabolic model to use for 13C-metabolic flux analysis (13C-MFA). The basic idea is to perform multiple tracer experiments with different isotopic tracers and then set one of these data sets aside to be used as validation data. The remaining data sets are then used to estimate fluxes using different metabolic models with increasing complexity. The hope is that the validation data will allow proper model selection, that is, selection of a metabolic model that is not too simple or too complex.

The authors are correct that model selection can have a significant impact on the fluxes that 13C-MFA produces, however, I disagree with the proposed approach for several fundamental reasons.**

We thank Reviewer 1 for a careful reading of the manuscript and for raising three important points, which all are central to the new proposed method. Since all of these three points concern different aspects of the same problem - and which all revolve around the same one solution - we start here with some initial general remarks, before we move into the specific point-by-point replies.

In brief, the three points address the proper choice of experimental validation data: how can one ensure that the independent validation data is both not too similar to existing data to constitute a trivial prediction (point 2), while at the same not too dissimilar to make an accurate prediction impossible (point 3), and how does choice of experimental tracer design come into this balancing (point 1)? We agree with Reviewer 1 that these three points were not addressed properly in the previous version of the manuscript. The reason for this was that the previous version lacked one important aspect: uncertainties of the prediction. With a method to calculate the uncertainty of the predictions in place, one can balance the "novelty" of the new experimental design, so that it is neither too similar nor too dissimilar. We have therefore used a previously developed general method for calculation of such uncertainties, and applied it into the field of EMU modelling, and into the updated version of the manuscript (Materials and methods, Section 4.4), where we now also describe and illustrate how this new method can be used to analyse the degree of novelty in the validation experiment design (Results, Section 2.4).

Let us add some more details regarding this new addition, before we move into the point-by-point reply, first i) regarding what this prediction uncertainty entails in general, and then ii) regarding how it can be used to avoid detect the possible problems raised.

i) The prediction uncertainty is essentially the confidence interval of the model predicted MIDs. This prediction uncertainty will depend on the uncertainty of the estimated fluxes, the model structure, and on the connection between the validation data and the estimation data. If the estimation data has sufficient information to determine some specific MID, the uncertainty of the MID will be small. In practice, this prediction uncertainty can be calculated e.g. using a methodology for prediction uncertainty that we presented in a general systems biology context in (Cedersund, FEBS J, 2012, 279(18):3513-27), and which is applicable for a general class of models, including EMU-models, even though the method has not been used in this field before. Shortly, the method generalizes the profile likelihood approach that already is widely used in EMU modelling for calculating the uncertainty of fluxes, to the uncertainty calculation of any model property, including an MID.

ii) The usage of these prediction uncertainties can be used to see whether the predictions are too novel or too similar. If the validation experiment is too novel, this means that there is no information in the estimation data to say something about that prediction. This will then be seen as a high prediction uncertainty. Specifically, for MIDs, such predictions which are too uncertain will have a confidence interval that is close to [0, 1](Fig B below, Fig 10B). If such predictions are seen, the validation experiment chosen is too dissimilar. In the opposite extreme, where the validation experiment will not produce any sufficiently novel data, this can also be seen in the prediction uncertainties. More specifically, if there is no new information in the validation data, the different model structures will produce the same predictions, and typically the same as the estimation data, i.e. the uncertainties will overlap (Fig 10A). The ideal situation is thus to have well-determined predictions (which we sometimes call core predictions), which are different from those in the estimation data (Fig 10C). We tested this for our EMU-example (Fig 11), and showed that we indeed had different predictions in the validation experiment (light blue bars), compared to all of the three tracer data used for estimation (red bars). In other words, for e.g. cis-aconitic M0 MID-data, the prediction uncertainty interval (light blue bar's error bar) is non-overlapping with all of the estimation data intervals (red bars' error bars), but that it is overlapping with the validation data (dark blue bar's error bar).

With these general remarks in place, let us now specifically comment on the three major concerns raised.

**Major concerns:

1) The authors completely ignore the most important step in 13C-MFA, which is the design of tracer experiments. In this step, the key objective is to select the most informative isotopic tracers and isotopic labeling measurements for a given metabolic network model. In this step, the metabolic model must represent the best current knowledge of the biochemistry of the cell that is studied, i.e. the model should be as comprehensive as possible to avoid any biases in the 13C-MFA studies. The approach proposed by the authors ignores the importance of proper tracer selection. If poor tracers are selected, then it doesn’t matter what method is used for model selection, the flux results will be poor. The authors must evaluate what impact good tracer selection and poor tracer selection has on their proposed approach.**

We fully agree with the reviewer that selection of tracers is a crucial part of 13C-MFA. We did not focus on tracer selection in this contribution since it is independent of model selection in the sense that, regardless of the tracer used, a proper model selection procedure should yield the model best suited to explain the available data. It is certainly true that, if in the tracer is poorly chosen, no model will provide accurate flux estimates; however, this a problem of that particular experiment, not a problem of the model selection procedure in general.

In response to the reviewer’s concerns, we have investigated the effect of using a poor tracer on the validation-based model selection procedure. A poor tracer results in uninformative MID data, which in the standard $\chi^{2}$-framework produces wide confidence intervals for the fluxes. With validation-based model selection, a poor tracer also yields wide confidence intervals for the predicted MIDs that are compared to the validation data, which in turn makes the model selection uncertain (as it should be; without informative data, we cannot hope to select models with any confidence).

In this example, we used data from the tracer [3-13C] Pyruvate as estimation data for the same TCA-cycle model that was used in the simulated ^13^C MFA example. This tracer is a good example of a poor tracer as the only labelled carbon atom in pyruvate is cleaved off and thus no isotope label ever enters the main part of the metabolic network. While the model can estimate this lack of labelling quite well, i.e. there are only a small differences between the purple MID data and the light purple model estimations, the estimated fluxes have very wide confidence intervals. If we then used this estimation to predict the MIDs for another tracer, in this example [U-13C] Glutamine, two things become apparent. Firstly, the prediction for the new tracer that corresponds to the best flux solution is quite poor (light orange bars). Secondly, since the flux estimates have very wide confidence intervals the predictions also have very large uncertainties. These large prediction uncertainties mean that the model predicts that the MIDs for the Glutamine tracer can have any value between 0 and 1, i.e. a completely uninformative prediction. We thus see that, for a poor tracer, the model predictions are very uncertain, and thus the comparison between model prediction and validation data is uninformative. In other words, comparing the model with validation data should not increase our confidence that the model accurately describes the metabolic system that is investigated. This demonstrates how one can use prediction uncertainty, to identify such problems, which has to do with a too informative tracer estimation data, or - alternatively - with a too novel validation data.

Finally, we will also want to mention that we would find the same large uncertainty in our model’s predictions if the model structure is unable to use the information contained in informative MID data (i.e. even with MIDs from a good tracer). This is why the choice of model structure is also important to consider when conducting 13C MFA.

**2) The proposed approach only really works if the validation data contains truly “novel” data that is independent of the other data. In reality, this can never be satisfied. How do you determine to what extent the validation data is independent? This is an important question that is never quantitatively addressed by the authors. The fact that a different tracer is used doesn’t automatically mean that the validation data is independent.**

Here the reviewer raises another excellent point. We fully agree that “novel” validation data is critical for validation-based model selection. In the simplest interpretation, this simply means that we should avoid the situation in Fig 10A (see above), where the estimation data and validation data are too similar, given the uncertainty in predictions. However, there are more subtle things to consider. Roughly speaking, one can say that the validation data is independent of the estimation data if it also depends on the underlying fluxes in a different way. Specifically for EMU systems, the notion of independence is nicely captured by the EMU basis vector concept: two experiments can be said to be independent if the tracers used produce linearly independent EMU basis vectors, which guarantees that the experiments give complementary information. This criterion can be used to ensure that the validation data is truly “novel”.

We have therefore now applied EMU basis vector analysis to the examples presented in the manuscript. This analysis has confirmed that the tracer experiments that generated the estimation and validation data have different basis vectors and are thus are indeed independent. We now also discuss this important issue in section 2.4, which is the new section added in response to Reviewer 1 (see above). **3) On the other extreme, the validation data must not be too dissimilar from the other data sets, or the validation data may not be predictable by any model. While this problem is briefly mentioned by the authors, is not fully addressed. This is a serious problem of the proposed approach. How do you ensure that the validation data is not too similar but also not too dissimilar compared to the other data? Unless the authors can provide some quantitative metrics that can be followed to quantify the degree of similarity and dissimilarity of the validation data, I am afraid that I cannot recommend the use of the proposed approach.**

As is mentioned in the general reply above, the prediction uncertainty method can address this point (Fig 10B). As discussed there, and in our response to point 1, the prediction uncertainty relates both to how well-estimated the fluxes are, and to how the model structure can use the information in the estimation data to make a well-determined prediction. This means that if the estimation data is uninformative, as in the example shown in our response to point 1, or if the model structure is not sophisticated enough to make the prediction, i.e., the model structure is missing some vital component of the “true system” that generated the data, the prediction uncertainty will be very large. In such situations, i.e. if it looks too much like in Fig 10B, the validation data is too dissimilar. The uncertainty of the predicted MIDs is a fully quantifiable metric, which also is connected to underlying statistics and p-values.

In summary regarding all these three main points, we understand why the reviewer feels that we had not addressed these points sufficiently in the main manuscript. To remedy this, we have now added the new theory of prediction uncertainty to the manuscript, and also provided an example in which we conduct the relevant analysis to determine the prediction uncertainty for the chosen model structure considered in the simulated ^13^C MFA example.

**Reviewer #2: The paper addresses the topic of model selection in metabolic flux analysis with isotope labeling data gathered under metabolic steady state conditions (termed MFA) when the labeling data error is largely unknown, but consistent across the data set. The authors compare different existing model selection approaches (SSR, first Chi2, best Chi2, AIC, BIC, validation) by means of simulated examples (polynomials, linear toy and nonlinear MFA systems) and apply them for the evaluation of a real data set in human epithelial cells. In the latter case, they found evidence for the pyruvate carboxylase as a necessary model component. The authors argue that model selection based on validation is a more reliable approach than SSR- and Chi2-based metrics in case of unknown data standard deviations. A conclusion about AIC/BIC is not drawn.

Overall, the work addresses a relevant, up to now underrepresented topic in the field of MFA. Some major issues concerning the precise aims/scope/results of the work need to be addressed. If done, the manuscript has the potential to become fit for publication in PLOS Comput Biol after some major revisions.

**Major Comments**
1. Model selection is always connected to a goal. The authors show examples for flux estimation, pathways inference and label prediction. Is the intention of model selection in this work directed towards all of these three categories? That this is not clearly stated is a weak spot of the manuscript; and it makes it somewhat hard to assess whether there is substantial evidence for the conclusions.**

This is an interesting question. We would argue that the three examples of flux estimation, pathways interference and, labelling predictions that are presented in the manuscript all are connected by the same underlying goal of identifying a suitable model for the metabolic system. These three topics are also highly interconnected with each other: only if the pathways inferred are correct will the fluxes be correct, and we argue that the best way to test and identify this model structure, is to use new and suitable labels in validation experiments. In other words, in either of the three above-mentioned goals, it is importance that that the user of the model is confident in that the model structure since this is connected all other predictions. The manuscript demonstrates that a validation-based approach can be used to identify a model structure that can make such reliable predictions, and that one of the added benefits is a robustness towards an unknown error in the magnitude of measurement uncertainty. Finally, with these things said, in practice, the most common reason to do EMU-modelling is to estimate fluxes. We have tried to revise the manuscript to make all of these points more clear.  **2. The presented results achieved across the examples, indicate that the performance of comparably cheap criteria (AIC, BIC) appear to be on par with the validation-based (VB) approach for all cases for which they are reported (Why do the authors calculate model selection AIC/BIC criteria for all, but the epithelial cell example?). Interestingly, this is not discussed. In addition, VB is computationally expensive. An overall comparison of criteria performances should be added incl. a discussion of the cost/benefit aspect.**

The reviewer points out an important aspect that was unclear in the previous version of the manuscript, concerning the performance of AIC and BIC. For the polynomial and linear examples, the dependence on errors in sigma could be seen already before (Fig S1-S2), but not in the simulated 13C MFA example, even though the dependency of course still is there (it is built into the method that errors in sigma leads to errors in judging model complexity, since sigma appears in the AIC and BIC formulas). To make this issue more clear also in the MFA example, we have expanded the range a bit in the new analysis (Fig S4), so that the dependency on sigma in AIC and BIC is seen. The reviewer also points out that the authors have not presented the AIC/BIC analysis for the epithelial cell example. This is correct and was simply an oversight on the authors' part. These results were not intentionally omitted and have been included in the revised version of the manuscript. Note that we do not know what is true result in this last analysis, based on real data. As the reviewer suggests, we have expanded the discussion to provide a more nuanced discussion of the cost/benefit aspects when choosing between the methods (see also Point #4 below).	**3. Following up Comment #2, the AIC is an approximation of leave-one-out cross-validation according to Gelman's Bayesian Data Analysis. Could then the AIC in theory not even be better than the proposed VB scheme?**

The reviewer notes that the AIC approach is an approximation of a leave-one-out cross-validation scheme and thus could in theory be better than the proposed validation-based approach. This is an interesting question and one that would require further investigation to answer comprehensively. However, the main problem with AIC in this context is that the AIC approach relies on the magnitude of the measurement uncertainty $\sigma$, and thus is at risk of selecting the wrong model structure when sigma is unknown (see answer to point #2 above).

Further the validation-based proposed in the manuscript differs from classical leave-one-out cross validation in that the validation data used in the proposed approach is generated from a different distribution (set of input/output measurements) than the distribution that generated the estimation data. Comparatively, in classical leave-one-out cross validation, the validation data is usually only a different realisation from the same distribution that generated the estimation data. In order to clarify this point, we have revised the Discussion to emphasize this difference for the reader.  **4. The Best Chi2 approach performs in some cases better than VB (in terms of fractions), in particular for the case of approx. correct and larger errors. Can the authors comment on this behavior? Together with Comments #1,2 above, this may find its way into a more fine-grained advice to modelers that apply model selection in cases of known vs. grossly known vs. unknown model errors rather than suggesting the consideration of VB per se (P19).**

The reviewer is correct in observing that the presented results might suggest that the “Best Chi2” method is equal to, or sometimes even more accurate than, the validation-based method. This specifically holds in the results presented for the simulated 13C MFA example (Figure 8). However, the apparent superiority of “Best chi2” in the simulated MFA example is mainly due to the limited range of the believed sigma ($\sigma_{b}$) presented (see point #2 above). For the results presented in the original submission, the authors only looked at a preliminary analysis of a wider range of the believed sigma, due to the computational load required for such an analysis. But in response to the reviewer’s concerns, we have decided to include the comprehensive analysis of a wider range of believed sigma values. The result of this extended analysis reconfirms the conclusions made in the manuscript, that “Best Chi2” is dependent on the magnitude of the believed sigma. This is as expected, since sigma appears explicitly in the chi2-formula. Therefore, in terms of a new and improved robustness towards an unknown magnitude of sigma - which is the main claim of the paper - the validation approach is still superior. Since sigma is unknown in MFA-modelling, and since we do not know at which degree of errors in sigma the problems appear, we argue that this is a critical advantage. Nevertheless, Reviewer 2 is correct in pointing out that a more nuanced comparison between the different methods can be done, and we have included such a comparison in the updated Discussion.	**5. Please add some references on the origins of the epithelial network model and on which basis the model variants are constructed. A network description directly attached to this manuscript would aid understanding and bridge between the thumbnail-sized pictures and the reaction names discussed in the text (see also below).**

The network model used for the epithelial cell example was not based on specific references but rather constitutes the most common reactions in and directly around the TCA-cycle, which is highly well-characterized in general biochemical literature. However, the authors agree with the reviewer that a model description would aid in the readers understanding, and we have therefore provided such a description in the revised supplementary material. Further, Full-size graphical networks for the seven model structures that are presented in Figure 7 are provided as .svg files in the supplementary material (in /OpenFLuxModels/ModelStoichiometryGraphs/). Also, PDF versions of these figures have been added for improved accessibility.

****Minor Comments**

1. Abstract: "These errors are often not known in practical examples." This statement suggests that it is standard that nothing is known about data errors in the field of MFA. This is certainly a disputable claim that may snub analytical chemists working in this field. Arguably, there is lots of work where approx. errors are formulated on well-grounded experience. Therefore, this reviewer suggests that the authors revise their statement appropriately.**

The authors agree with the reviewer’s suggestion and have revised the abstract and mansucript accordingly.  **2. P4 "s does not account for experimental bias, such as deviations from metabolic steady-state that always occur in batch cultures." This statement is difficult because if the MSS considerably questioned, applying MFA is doubtful. Moreover, wouldn't repetition of the experiment help to (un)cover such deviations at metabolic level?**

This is another interesting topic, about which there is much to say. Shortly, some experimental problems could be detected by repetitions of the experiments, but some would not be. For instance, if the biological system is not in steady state, but in the middle of a long transient, a repetition of the same experiment would give the same result each time. In such cases, one would need repeated experiments at other time-points, to discover - but not solve - the problem. However, in batch cultures, this is always the case, and the question is therefore if it is ever a meaningful assumption to assume that a traditional steady-state MFA-model provides a useful approximation of data from a batch system. Such a model will, by definition, be wrong, but it may still provide sufficiently reliable predictions. A similar situation appears e.g. if the studied cells have a heterogeneity, i.e. then the traditional assumption of an average cell does not hold. To test whether the classical assumptions of steady-state metabolism in an average cell is good enough to produce reasonable predictions, validation tests are important.

This is a big topic, and a comprehensive analysis is a bit out of scope in this manuscript. We have tried to reformulate the text around the sentence in the introduction, mentioned above, and we also return to the topic shortly in the end of the updated Discussion.

**3. The authors speculate about the approaches that are applied in the MFA domain for model selection. Indeed the procedure described in (Antoniewicz 2018) may be interpreted as First Chi2 approach. The iterative scheme sketched in (Dalman 2016) points more to the use of the SSR/Chi2 as a means for model validation/testing and in this** sense **would have been correctly applied. Generally, it would improve clarity if the authors introduce the differences between model validation/testing and model selection in the introduction, rather than mentioning it on the last page, and take care distinguishing these two traits in commenting the literature.**

We agree with the reviewer that it is important to cover the literature as correctly as possible. It has, in our experience, been hard to exactly assess how other research groups have worked, based on their publications, since the exact steps of model development and selection are only rarely explicitly mentioned. Regarding Dalman 2016, it clearly outlines an iterative approach, just as Reviewer 2 points out. However, in our reading it is not clear that this constitutes something else than First Chi2, since there appears to be neither a design of new experimental data used for validation, nor an explicit sub-division of data into estimation and validation data. In any case, we refer to this particular reference as an example of a manuscript that describes iterative improvement of a model until an acceptable model is found.

Also, just as Reviewer 2 suggests, we have pointed out the difference between model selection and model testing earlier in the manuscript: it now appears on the first page in the Results section.

**4. P6: "For all examples herein, data from distinct model inputs is used for validation." Does this mean data from tracer experiments with different tracer species? Please clarify.**

Yes, the statement refers to that for the 13C MFA examples the validation data comes from a different tracer. This statement have been revised to offer further clarity for the reader.  **5. Sec. 2.1: The motivating example, although being a simple illustration to unfamiliar readers, is clearly outside the main story line. Thus, this reviewer suggests to consider shifting the example to the supplement, and keep focused on labeling systems.
Also, here the difference between model validation/testing and selection becomes evidently mixed (see also #3): the most simple and most complex polynomials will certainly fail in a model validation/testing step.**

The authors feel that the motivating polynomial example is relevant for introducing the reader to the concepts presented in the paper in the familiar and well-established context of choosing the right polynomial. This problem has several well-established features which we believe serves as a good basis for the rest of the manuscript. Further, we do not fully agree with the sentiment that this example confuses the concepts of model validation/testing and model selection. To remedy such possible confusion, we have tried to make this difference more clear in the text.

**4. P11: Please report the number of resamplings in the main text, rather than just in the caption of Fig. 8.**

The text has been revised to properly report the number of resamplings for the simulated 13C MFA example.  **6. P13/Fig. 9: Please report the full name of the reactions discussed to "link" the results better to the metabolic network context (see also Major Comment #5).**

Figure 9 and the corresponding text have been revised to properly state the full name of the relevant reaction enzymes.  **7. Sec. 2.4 Please report also AIC/BIC numbers (see also Major Comment #2).**

The AIC and BIC results for the epithelial cell example have been included in the revised version of the manuscript. **8. P15: "suggesting that fatty acid synthesis indeed occurred". Please clarify whether there is potentially another route than FA synthesis for label entering this compound (see also Major Comment #5).**

13C atoms can (and do) enter LPC 16:0 also in the glycerol moiety, which is evident from the marked M+3 seen in both LPC 16:0 and glycerol-3-phosphocholine. However, the mass isotopomers M+4 and higher of LPC 16:0 can only derive from the acyl chain, indicating that fatty acid synthesis must occur. We have revised this sentence to clarify.

**9. Fig. 11: Please add error bars to the mean MIDs.**

We have now added error bars to this figure. **10. P17: "correctly predicting" brings up the question on how correct, correct is. The more qualitative wording used on P15 "reasonable good fit" appears to be better suited here.**

The authors agree with the reviewer and have revised the manuscript accordingly.  **11. P18: "In practice, the magnitude or scaling-error of is not necessarily the same for all data points, and the results herein indicate that the new validation-based is a good choice also in such situations". To the understanding of the reviewer, this has not been tested. Please comment.**

This is an important point, and one that we - in fact - do test, at least to some extent. In section 4.5, we describe how the simulated data is generated. At the end of this section, we describe how the values for the believed sigma $\sigma_{b}$ are generated from a distribution, such that $\sigma_{b}$ is not identical for each sample, but rather has the desired approximate magnitude. To clarify this earlier in the manuscript a short comment regarding this has been added to the Discussion. **12. For clarity, please use consistent names for the different test cases throughout text and figures, e.g. "simulated 13C-MFA model example" in Fig 7+8+9, or "cultured epithelial cells" in Fig. 10**

The text has been revised to be more consistent when referring to the different examples.  ****Typos/Wording****

**P2 This problem is central
P3 Correct system hypothesis --> System hypothesis
P4 approached --> approach; passes --> pass; are often below --> are often reported to be below (add reference?); former alternative leads to high uncertainty --> former alternative may lead to high uncertainty
P9 simulate data form (--> from) 6 distinct input vectors
P14 tested --> applied
P23 check notation: M0 model with theta0 parameters is selected for data generation, with M0=M7 in case of the polynomial example, M0=A3 in case of ... etc.**

All these typos/wording concerns have been revised in accordance with the reviewer’s comments.
