# Supplementary material for: Validation-based model selection for 13C metabolic flux analysis with uncertain measurement errors
Source: PLoS Comput Biol. 2022 Apr 11;18(4):e1009999. doi: 10.1371/journal.pcbi.1009999 (PMC9022838; doi:10.1371/journal.pcbi.1009999)
Supplement: Attachment — Submitted filename: Response_to_reviewers_2022_01.docx [file pcbi.1009999.s012.docx]

**Reviewer #2: The minor comments below are mainly related to sharpen statements that otherwise risk being misunderstood by the target community. Since line numbers are missing, page numbers are given.**
We thank reviewer #2 for having so diligently gone through the manuscript and we agree that the suggested revisions will help clarify the statements made in the manuscript. We have therefore revised the manuscript in large part in accordance with the reviewer’s comments and added clarification where needed. The specific revisions concerning each comment is specified below.

**Minor Comments
1. Reply to Response to Major Comment 5:
While the list given in the SI about model variants is clearly useful as an overview, this way is clearly insufficient for reproducing results or even a simple model simulation step. Please take care to adhere to the reporting standards established in the field.**

We agree that it is very important for scientific integrity to be able to completely reproduce all results. Therefore, all files required to reproduce the results have always been included in the supplementary material, this comment most likely means that the files were difficult to find. These supplementary files include files needed to run all required model simulations and all specific model definitions. The ready to run files are structured in folders and the model definitions are structured in accordance with the software OpenFLUX v2, which is widely used in the field. To make these files easier to find, we have added the following text both at the start of Materials and methods and under section 4.8. “All details regarding implementations, including both text documents and all files needed to reproduce all results, are available both as supplementary material at the journal homepage and at: <https://gitlab.liu.se/nicsu70/val-based-model-selection_13cmfa> ”

**2. Reply to Response to Major Comment 4 and Minor Comment 1:
Abstract, Summary, P15: This reviewer agrees that estimating the precise true error is hardly possible. Indeed, results presented indicate that gross mis-characterization of these errors leads to non-robust model selections, which in turn CAN lead to errors in flux estimates. But, in standard use-cases, such as E. coli and GC-MS, the order of the errors in the labeling data is indeed well characterized. Here, the results presented do indicate that if the true error is approximately correctly known, traditional methods work fairly well *and* are computationally efficient. Thus, validation-based model selection is useful in cases for which the magnitude of the errors is unknown or a gross mis-characterization is suspected. Please be precise here and adequate in the conclusions.**

We agree with the reviewer that the statements in the Abstract, summary and on P.15 are a bit too general. We have therefore revised these statements to more precisely specify that the validation-bases approach is most useful in cases where the magnitude of the measurement error is unknown.  **3. Reply to Response to Minor Comment 2:
Certainly, modeling assumptions are always idealizing the truth. The fundamental principles and experimental requirements for 13C MFA are well-known and -established, and it was not intended to scrutinize them here. The point is that it is dangerous to think about MID error models as means to cover deviation from metabolic stationarity. Labeling error models are, as the authors specify, assumed to be normally distributed without bias. But whether errors introduced by metabolic instationarity are also of this type is unclear. Please clarify, to circumvent the naive conclusion that an increase in error could "heal" metabolic instationarity.**

We agree with the reviewer that the current text could suggest that deviations from the metabolic steady state could be mended by increasing the error. The text has been revised to clarify that simply increasing the error does not necessarily compensate for such other experimental problems.  **4. Reply to Response to Minor Comment 3:
Since the underlying principles of incremental model updating used in Dalman 2016 seems to be not perfectly clear, this reviewer suggests replacing or amend this cite on P3 by 10.1038/s41596-019-0204-0, where the procedure is outlined.**

We agree with the reviewer and have replaced the reference on P. 3 with the suggested reference.  **5. Abstract:
The notion of prediction uncertainty should be formulated more precisely, i.e. labeling prediction uncertainty, since the constraint-based modelling community often uses the term "flux prediction".**

We agree with the reviewer that specifying that the presented approach is used for quantification of the labelling prediction uncertainty. The Abstract have been revised accordingly.  **6. P4: "By quantifying ... core prediction," is hard to understand without further background.
It may be better so state why prediction uncertainty is important and validation data should be chosen wisely, instead of explaining how this is done here. Later, when the prediction and inference uncertainties are discussed, a statement is warranted what such "core predictions" are. Also, the authors may comment why MCMC is preferred over the standard PL-based approach for flux confidence bounds, whereas for prediction uncertainty the PL-based approach and not a standard MC approach is taken.**

We have removed mentioning of the term core predictions from the introduction, as suggested. Regarding the MCMC vs PL: we did not mean to suggest that MCMC is the preferred method. Both methods were employed and are therefore described in methods. While all results could be achieved with the PL-analysis, MCMC was used at some points in the interest of computational efficiency. Sec. 4.4 has been revised to clarify that profile likelihood is the primary method employed.  **7. P13: "This theory says ... goes to infinity."
This is not generally true. An important assumption is stated later in the paragraph, but in a rather disconnected manner. Please put it in direct context. For instance, in case the data is informative and no structural non-identifiabilities are present, theory says ...**

We agree with the reviewer that the assumption made for this specific statement was not clearly presented in connection to the statement itself. The paragraph has been revised such that the assumption is put in direct context of the initial statement. **8. P13: "and the results will still hold as long as the same difference between metabolites holds also for sigma_r".
Unclear, please revise.**

This statement has been revised to clarify that the presented results will also hold in cases where there is a difference in magnitude of the believed error between different subsets of the data. As long as the same difference is also present for the true measurement error. In other words, if data for one metabolite is substantially more uncertain than the data for another metabolite, $\sigma_{b}$can reflect this difference in uncertainty, and the results will hold as long as the same difference is present in $\sigma_{r}$.

**9. P14: "compared to methods for mechanistic modeling, where new distributions can be used for the analysis".
Unclear, please revise.**

This statement has been revised to clarify that the presented methods for mechanistic modelling differ from statistical methods of validation in that the presented methods can be used when validation data and estimation data are generated by different distributions. Compared to statistical methods where the validation data and estimation data always comes from the same distribution.  **Typos:**

The typographical errors have been revised were needed.

**P3: model structures that pass the Chi2 test.; the underlying errors**
For both of these cases the singular form is correct (i.e. model structure and underlying error). In the first sentence we refer to a single model structure and in the second sentence the full sentence read “Second, the $\chi^{2}$-test can be unreliable in practise since the underlying error model is often not accurate”.

**P6: First Chi2 (without ")
P9: can be used for; for this new method in 13C MFA; does not contain any new information (instead provide)
P13: offer a pragmatic way forward
P14: of these results is that also
P15: on errors in sigma_b
P18: Sect. 4.4 check tense
References: Check duplicates (e.g. Aitchison)**
